# Evaluating the Effects of Exercise on Inflammation Markers in Musculoskeletal Pain: A Systematic Review and Meta-Analysis

**DOI:** 10.3390/sports13060168

**Published:** 2025-05-29

**Authors:** Chi Ngai Lo, Nicole Elizabeth Jing Wen Wong, Shina Ho, Elicia Jia Hui Ang, Bernard Pui Lam Leung

**Affiliations:** 1 Family Care Physiotherapy Clinic, 154 West Coast Road, 85 West Coast Plaza, 01-86, Singapore 127371, Singapore; 2 Health and Social Sciences, Singapore Institute of Technology, 10 Dover Drive, Singapore 138683, Singapore

**Keywords:** musculoskeletal pain, exercise, inflammation, cytokines

## Abstract

This systematic review and meta-analysis aimed to investigate the effectiveness of exercise interventions in regulating inflammatory biomarkers among individuals with musculoskeletal pain. A comprehensive search of MEDLINE, CINAHL, Web of Science, Cochrane Library, and Google Scholar was conducted from inception to November 2024. Only randomized controlled trials (RCTs) published in English that examined the effects of exercise on inflammatory markers—such as C-reactive protein (CRP), interleukins (ILs), and tumor necrosis factor-alpha (TNF-α)—were included. Twenty-three RCTs involving 1128 participants met the inclusion criteria. Meta-analysis of four studies indicated that isokinetic exercise significantly reduced CRP (MD = −0.40, 95% CI: −0.44 to −0.36, *p* < 0.01, I^2^ = 0%), IL-6 (MD = −1.59, 95% CI: −2.61 to −0.56, *p* < 0.01, I^2^ = 97%), and TNF-α (MD = −4.24, 95% CI: −5.13 to −3.36, *p* < 0.01, I^2^ = 90%) levels compared to general exercise. These findings suggest that exercise, particularly isokinetic exercise, may reduce systemic inflammation in patients with musculoskeletal pain and provide therapeutic effects beyond mechanical improvement. The review followed PRISMA guidelines and was registered on PROSPERO (CRD42024500081).

## 1. Introduction

Musculoskeletal pain is one of the most prevalent health conditions, affecting individuals across all age groups and significantly impacting daily function and quality of life (QoL). It encompasses a range of disorders, including osteoarthritis (OA), rheumatoid arthritis (RA), chronic low-back pain (CLBP), fibromyalgia (FM), and other degenerative or inflammatory conditions affecting the joints, muscles, and connective tissues. The global burden of musculoskeletal pain is substantial, leading to high healthcare costs, loss of productivity, and increased disability rates [1,2]. Managing this condition requires a multimodal approach that includes pharmacological treatments, lifestyle modifications, and physical interventions. Among these strategies, exercise therapy has emerged as a cornerstone for pain management and rehabilitation [3,4].

The role of exercise in alleviating musculoskeletal pain is well documented; beyond its mechanical and neuromuscular benefits, exercise is increasingly recognized for its systemic effects, particularly in regulating inflammation [5,6]. Recent research has highlighted the potential of exercise to modulate inflammatory processes, which are often implicated in the pathophysiology of musculoskeletal disorders [7]. Chronic inflammation is a key contributor to pain and tissue damage in musculoskeletal conditions such as OA, RA, and tendinopathies. A range of inflammatory markers and related substances, including interleukins (ILs), tumor necrosis factor-alpha (TNF-α), and C-reactive protein (CRP), have been frequently reported as elevated in these populations [8,9,10].

In addition, immunomodulatory cytokines such as IL-1β, IL-10, IL-17, IL-2, and interferon-gamma (IFN-γ) are also frequently assessed in clinical trials to reflect the status of inflammation. For example, IL-1β, IL-17, and IFN-γ are pro-inflammatory cytokines. Elevated levels of these substances are commonly observed in joint inflammation, cartilage degradation, chronic pain, autoimmune diseases, and various forms of arthritis [11,12,13]. On the other hand, IL-10 is an anti-inflammatory cytokine that inhibits the production of pro-inflammatory mediators, and higher IL-10 levels are associated with reductions in chronic inflammation [14]. IL-2 is a regulatory cytokine that helps modulate immune activity and maintain inflammatory balance [15].

In musculoskeletal pain and arthritis, while conventional treatments such as nonsteroidal anti-inflammatory drugs (NSAIDs) and disease-modifying antirheumatic drugs (DMARDs) aim to control inflammation, they often come with potential side effects and limited long-term efficacy. Exercise, as a non-pharmacological intervention, presents a promising alternative or complementary approach. It is hypothesized that regular physical activity influences inflammatory pathways by modulating immune responses, reducing oxidative stress, and enhancing anti-inflammatory cytokine release. However, despite the increasing interest in exercise-induced immunomodulation, the exact mechanisms by which different types of exercise influence inflammatory biomarkers remain unclear [7,16,17]. Understanding the relationship between exercise and inflammation is critical for optimizing rehabilitation strategies for individuals with musculoskeletal pain. Aerobic exercise, resistance training, isokinetic training, and core stabilization exercises have been investigated for their impact on inflammatory cytokines, but there remains a lack of consensus on which exercise modalities are most effective in reducing inflammation-associated pain [7,18].

Exercise is increasingly recognized for its systemic effects, including modulation of inflammatory pathways, and is hypothesized to exert both pro- and anti-inflammatory influences depending on context, intensity, duration, and targeted tissues [16,19,20]. Previous studies have examined the effects of exercise on inflammatory responses in healthy individuals, patients with metabolic syndrome, and postmenopausal women. For instance, Cerqueira et al. (2020) found that high-intensity exercise promotes the production of immune molecules such as CRP, IL-6, and IL-10, with more pronounced effects compared to moderate-intensity exercise [20]. Particularly for IL-6, its systemic elevation has been closely linked to eccentric and concentric exercise, highlighting its role as a contraction-induced myokine with metabolic and immunological functions [21]. Alizaei Yousefabadi et al. (2021) reported a significant reduction in TNF-α, CRP, and IL-8, along with an increase in the anti-inflammatory cytokine IL-10, after 12 weeks of exercise in patients with metabolic syndrome [22]. In postmenopausal women, exercise training was found to significantly reduce IL-6, TNF-α, and CRP while increasing adiponectin, a molecule that reduces both oxidative stress and serve as an anti-inflammatory protein [23]. Despite these findings, the direct impact of exercise on inflammatory markers in musculoskeletal pain populations remains an area of active investigation.

The primary objective of this review is to evaluate the effectiveness of exercise, via structured and time-limited exercise interventions, in altering inflammatory biomarker levels in individuals with musculoskeletal pain and to determine whether these changes correlate with clinical improvements in pain and function. By employing findings from randomized controlled trials (RCTs), we aim to study the variability in inflammatory marker levels in patients with musculoskeletal pain in response to various types of exercise interventions.

## 2. Materials and Methods

### 2.1. Protocol and Registration

This review was conducted according to the Preferred Reporting Items for Systematic Reviews and Meta-Analyses methodological guidelines [24] and the *Cochrane Handbook* [25], with PROSPERO registration number CRD42024500081. The study selection process is based on strict inclusion and exclusion criteria, ensuring the inclusion of high-quality RCTs that specifically assess inflammatory biomarker outcomes in response to exercise interventions. This review also employs meta-analytical techniques to quantify the effects of exercise on inflammation, providing a robust synthesis of available data [25].

### 2.2. Search Strategy

A search was conducted in MEDLINE (via PubMed), CINAHL, Web of Science, Cochrane Library, and Google Scholar (supplementary searches only) according to the search strategy listed in Appendix A. Boolean logic was applied where necessary to optimize search results. Google Scholar was used for supplementary searches to identify additional relevant studies. Manual searches of previously published reviews and the reference lists of retrieved studies were also performed. The literature search covered studies from inception to 15 November 2024, with inclusion restricted to English-language publications.

### 2.3. Inclusion Criteria

This systematic review included only RCTs involving patients with musculoskeletal pain, such as joint pain, arthritis, neck and back pain, or tendinopathy. Eligible interventions were structured, time-limited exercise programs, including aerobic training, resistance training, combined protocols, or other specific active exercise regimens. The primary outcomes were levels of inflammatory biomarkers and cytokines, including CRP, ILs, and TNF-α, matrix metalloproteinase (MMP), and other related immune molecules.

Trials were excluded if the interventions included diet, medication, or other passive treatments exclusively in either the experimental or control group only, making it difficult to reflect the effects of exercise. Studies with uncontrolled long-term lifestyle physical activity or community-based exercise programs, case–control studies, and observational studies were excluded. Studies were excluded if the subjects were post-surgical patients because one could not ascertain if the inflammatory factors were from musculoskeletal pain or surgery. Additionally, studies without outcomes related to inflammatory markers were also excluded.

Database search records were copied to the online platform Rayyan (https://www.rayyan.ai/, accessed on 10 December 2024) for duplication removal and study selection. Two reviewers screened all articles independently for inclusion and exclusion criteria followed by quality appraisal after study selection. Other reviewers contributed to further opinions for discussion where there were disagreements.

### 2.4. Data Extraction

Data were independently extracted by each reviewer for the demographic information of the subjects, their main diagnosis, and the specific inclusion and exclusion criteria applied. Detailed information regarding the intervention, including the type and frequency of exercises, was also collected. Furthermore, outcome measures at baseline and follow-up time points were extracted. For studies sharing similar outcome measures, the software Review Manager, version 5.4 (The Cochrane Collaboration, London, UK, 2020), was used to pool the result data with formal meta-analytical techniques for analysis. Mean difference (MD) was used as the effect measure for outcomes reported with the same unit or scale, while standardized mean difference (SMD) was applied for outcomes measured using different scales across studies. The 95% confidence intervals (CIs) and heterogeneity (I^2^) were calculated to quantify the effect size and consistency of the results.

### 2.5. Risk of Bias Assessment and Summary of Evidence

The included studies were assessed for risk of bias using the Cochrane risk-of-bias tool for randomized trials—version 2 (ROB2) tool [26]. Five domains are included in the ROB2 as bias arising from the randomization process, bias due to deviations from intended interventions, bias due to missing outcome data, bias in measurement of the outcome, and bias in selection of the reported result. Two reviewers independently assessed each included study, and any disagreements were resolved through discussion or consultation with other reviewers. The risk-of-bias judgment for the RCTs was considered as low risk of bias, some concerns, or high risk of bias in each domain [26].

After conducting the meta-analysis, the quality of evidence for each outcome was further assessed using the Grades of Recommendation, Assessment, Development, and Evaluation (GRADE) framework. The justifications of GRADE are based on potential study limitations, the consistency of effects, the precision of results, and the relevance (directness) of evidence, providing a comprehensive appraisal of the pooled evidence’s quality [25]. According to these domains, the GRADE system can justify the certainty or confidence in the evidence as a continuum of four categories, ranging from high, moderate, low, and very low [27].

## 3. Results

**Figure 1:**
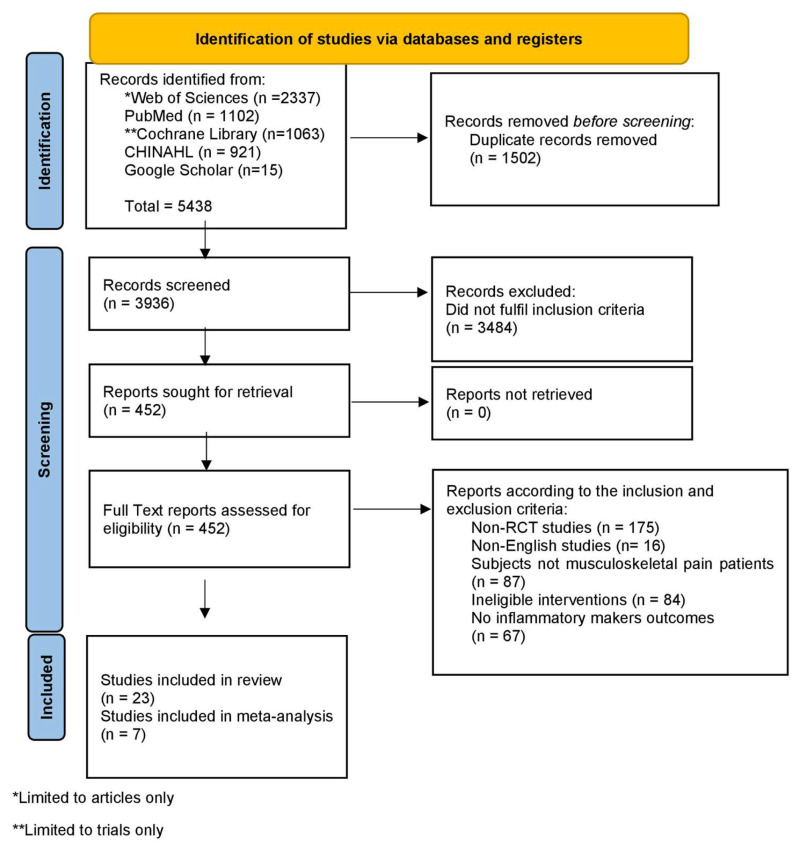
PRISMA flowchart.

The database search initially retrieved a total of 5438 records. Of the 3936 records screened after duplicate removal, 3484 were excluded at the title and abstract level primarily due to study design (e.g., non-RCTs, reviews, protocols), irrelevant populations (e.g., non-musculoskeletal conditions), or interventions not involving structured exercise. Among the 452 full-text articles assessed for eligibility, exclusions were made for reasons including non-randomized design (n = 175), use of non-English language (n = 16), populations not experiencing musculoskeletal pain (n = 87), ineligible interventions (n = 84), and absence of inflammatory marker outcomes (n = 67). These decisions followed predefined eligibility criteria and were made through independent manual review. No AI-assisted tools were used during the screening or exclusion process. After screening and applying the inclusion criteria, 23 RCTs were selected for analysis in this systematic review (Figure 1) [28,29,30,31,32,33,34,35,36,37,38,39,40,41,42,43,44,45,46].

In total, the included studies involve 1128 subjects, with an age range of 18 to 80 years old. The characteristics of the included studies are shown in Table 1 and Table 2.

Among the included studies, six articles focused on patients with OA, most of which implemented resistance training, isometric, or whole-body electromyostimulation (WB-EMS) as primary interventions. Three studies addressed RA and utilized a range of modalities including aerobic walking, Tai Chi, and neural mobilization to manage systemic inflammation and joint stiffness. Two studies involving fibromyalgia adopted moderate-intensity aerobic exercises such as Nordic walking combined with stretching or strengthening. For chronic low-back pain and non-specific back pain, five studies employed core stabilization exercises, isokinetic trunk training, or McKenzie methods. Four studies targeted chronic neck pain, primarily using cervical isometric or resistance band exercises to improve posture and reduce pain. Two studies addressed tendinopathy, where eccentric and isokinetic loading were the dominant strategies. Lastly, one study on axial spondyloarthritis implemented a combined protocol of WB-EMS and isometric squats.

Across the 23 included RCTs, a diverse array of inflammatory biomarkers were assessed to evaluate the effects of exercise interventions. The most frequently measured markers were CRP (reported in 10 studies), TNF-α (12 studies), and IL-6 (10 studies). Several studies also examined IL-1β, IL-8, and MMP-3 as indicators of localized inflammation or tissue remodeling. In addition to pro-inflammatory cytokines, anti-inflammatory and regulatory markers such as IL-10, IL-2, IL-4, and IFN-γ were measured in four to six studies, providing insights into the immunomodulatory effects of exercise. Less commonly reported markers included Eotaxin, IP-10, PTX3, and resistin, which were explored in a small number of trials. A few studies also measured surrogate inflammatory indicators like erythrocyte sedimentation rate (ESR) and rheumatoid factor.

The included studies varied in follow-up periods, ranging from assessments conducted immediately after the intervention to those extending up to six months post-intervention. The majority of studies had follow-up periods between 4 and 12 weeks.

### 3.1. Risk of Bias Assessment

**Figure 2:**
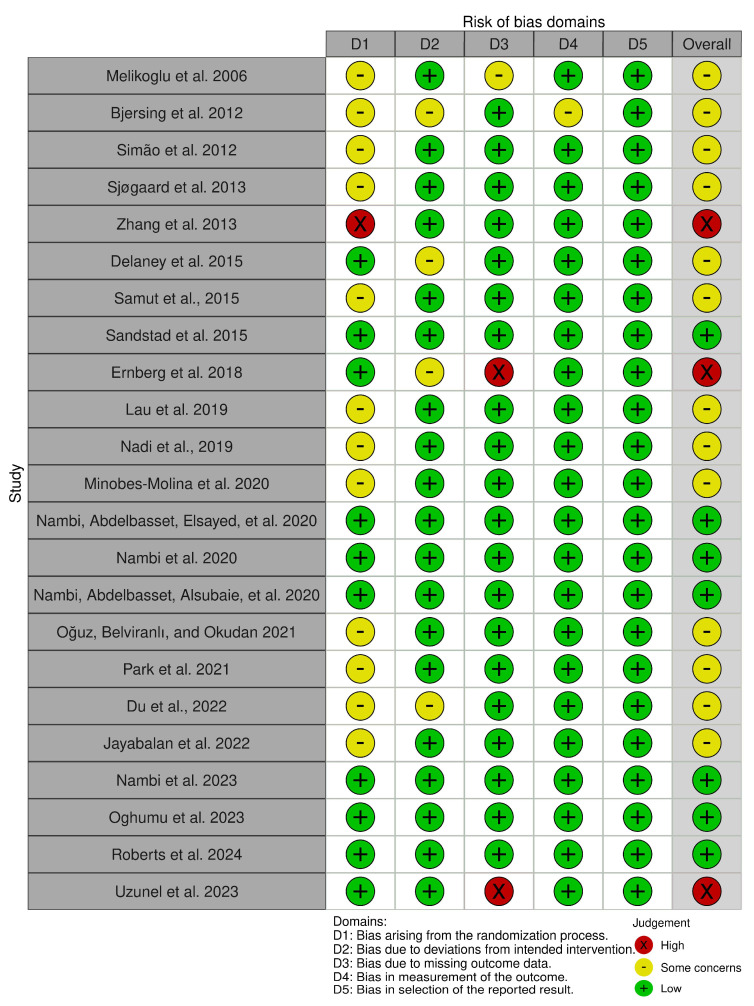
Risk of bias assessment.

**Figure 3:**
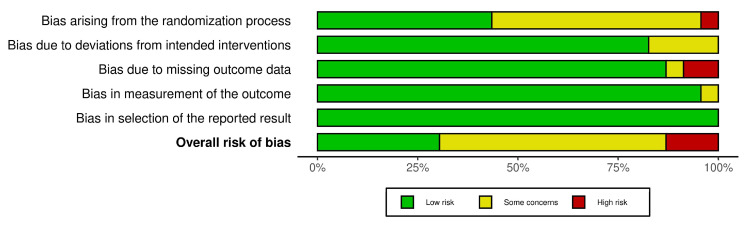
Risk of bias summary.

This systematic review assessed the risk of bias solely for biomarker outcomes, excluding other clinical outcomes. This limited approach may have introduced variability in the assessed risk of bias and study quality, as incorporating other clinical outcomes could have yielded different evaluations. Overall, 30.5% (7 out of 23) of the included studies were considered as having a low risk of bias, 56.5% (13) had some concerns, and 13% (3) were deemed to have a high risk of bias (Figure 2 and Figure 3).

### 3.2. Meta-Analysis

Two sets of meta-analysis were conducted with random effects to pool MD for the studies sharing the same scale of outcome measures, to pool SMD for those with different scales with 95% Cis, and to estimate heterogeneity (I^2^, %).

**Figure 4:**
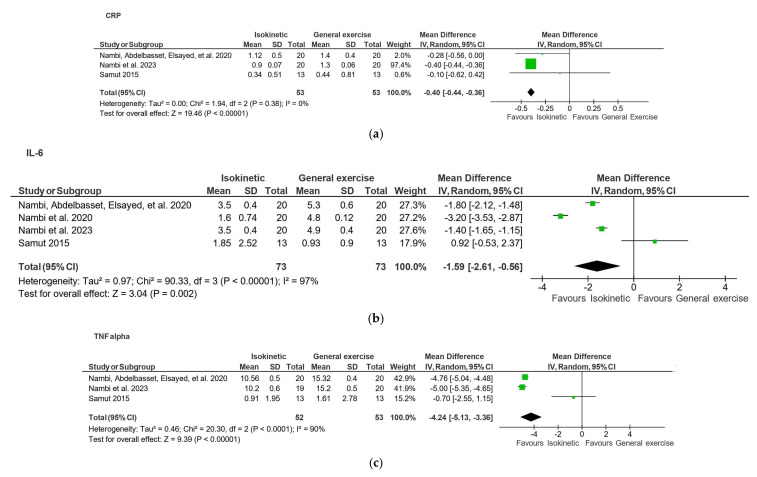
(**a**) A forest plot of the effect of isokinetic exercise and general exercise on CRP levels. (**b**) A forest plot of the effect of isokinetic exercise and general exercise on IL 6 levels. (**c**) A forest plot of the effect of isokinetic exercise and general exercise on TNF alpha levels.

From four included studies (two on OA and two on chronic back pain) [34,39,41,45], isokinetic exercise was found significantly more effective in reducing CRP levels than general exercise at 4–6 weeks follow-up, with an MD of −0.40 (95% CI: −0.44 to −0.36, *p* < 0.01, I^2^ = 0%) (Figure 4a). Similarly, isokinetic exercise led to greater reductions in IL-6 levels (MD −1.59, 95% CI: −2.61 to −0.56, *p* < 0.01, I^2^ = 97%) and TNF-α levels (MD −4.24, 95% CI: −5.13 to −3.36, *p* < 0.01, I^2^ = 90%) compared to general exercise (Figure 4b,c).

**Figure 5:**
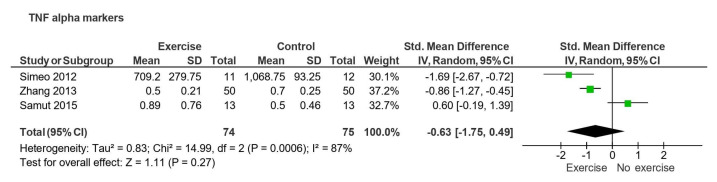
Forest plot of effect of exercise on TNF levels.

Three studies on patients with osteoarthritis compared active exercise to the control for TNF-α expression [30,32,34] with follow-up periods ranging from 4 to 12 weeks. The results showed no statistically significant effect (SMD = −0.63, 95% CI: −1.75 to 0.49, *p* = 0.27) and high heterogeneity (I^2^ = 87%) (Figure 5).

### 3.3. GRADE Recommendations

The strength of evidence according to GRADE criteria generated by the GRADEpro website (https://www.gradepro.org/ accessed on 10 January 2025) is listed on Table 3.

According to the results of the meta-analysis, a high GRADE recommendation supports the effects of isokinetic exercise in significantly reducing CRP levels in patients with musculoskeletal pain compared with general exercise. However, the overall quality of the GRADE recommendations on the effect of isokinetic exercise in reducing TNF-α and IL-6 levels in patients with musculoskeletal pain compared with general exercise was considered low. For osteoarthritis, the GRADE recommendation was rated as very low quality, suggesting that exercise has no significant effect on TNF levels compared with the control, according to the meta-analysis results.

## 4. Discussion

We conducted a systematic review and meta-analysis to evaluate the effects of exercise on inflammatory markers in individuals with musculoskeletal pain. Over the last decade, researchers have performed similar reviews on the effects of exercise on inflammatory response in healthy individuals [20], patients with metabolic syndrome [22], and postmenopausal women [23].

The results from Cerqueira et al. (2020) suggest that exercise has a significant acute effect in promoting CRP, IL-6 and IL-10, with higher effects from intense exercise than moderate exercise [20]. In patients with metabolic syndrome, the results presented by Alizaei Yousefabadi (2021) [22] demonstrate a significant reduction in TNF-α, CRP, and IL-8 but an increase in IL-10 after a 12-week follow-up period. Khalafi, Malandish, and Rosenkranz (2021) reported that exercise training significantly reduced IL-6, TNF-α, and the acute-phase protein CRP and increased adiponectin when compared with controls in postmenopausal women [23]. Their included studies had follow-up periods ranging from 8 to 24 weeks.

Our review suggests that high GRADE level evidence indicates that isokinetic exercise is significantly more effective than general exercise in reducing CRP levels in patients with musculoskeletal pain. For IL-6 and TNF-α levels, similar effects were observed, but the level of evidence was low. These findings are based on a 4–6-week follow-up period. In contrast, very low GRADE level evidence suggests that exercise has no significant effect on TNF expressions in patients with OA.

There is a strong biological linkage between CRP and IL-6. When inflammation is triggered, IL-6 is released into circulation and stimulates the production of CRP and other acute-phase proteins in the liver. As a result, CRP levels in plasma typically rise in parallel with IL-6 concentrations during acute inflammation [47]. However, the differences in our findings primarily reflect variations in research quality and consistency among the included studies. The meta-analysis on CRP included three RCTs with consistent results and a low risk of bias. In contrast, although IL-6 showed a similar direction of effect, the associated meta-analysis involved four RCTs with substantial heterogeneity (I^2^ = 97%) and wide confidence intervals, resulting in a low-certainty GRADE rating. The discrepancy is due to differences in specific research quality and evidence grading rather than biological mechanisms.

The included studies featured a variety of exercise interventions, including aerobic exercise, strengthening exercises, balance exercises, joint mobility exercises, and high-intensity exercises. In contrast, the control or comparison groups received low-intensity exercise, general non-specific exercise, relaxation therapy, joint mobilization exercises, or no intervention (see Table 2). To ensure a fair comparison based on the principles of population, intervention, control, outcome, and follow-up period, we selected specific studies with relatively similar characteristics so only two sets of meta-analyses were established. Future research on the effects of exercise may need a consensus on a standardized comparison intervention, allowing for more precise evaluation of specific exercise effects.

Analysis revealed distinct correlation patterns between biomarkers and the severity of clinical symptoms. In patients with RA, CRP was reported to be significantly correlated with tender/swollen joint count and disability (r = 0.46–0.80) by Du Liang and Guo (2022) but the sample size was small (n = 20) [44]. In sciatica patients, several longitudinal studies reported moderate positive correlations for serum (r  =  0.629) and biopsy (r  =  0.65) TNF-α with pain level; severe pain (VAS > 4) was also shown to be significantly associated with increased high-sensitivity CRP levels (adjusted OR  =  3.4 (95% CI, 1.1 to 10) [48]. In contrast, TNF-α, IL-6, and CRP levels were reported to have no or inconclusive evidence of correlation with pain in patients with chronic low-back pain and chronic whiplash-associated disorder [8]. Therefore, the levels of biomarkers maybe useful outcome measures to reflect the effects of exercise in certain musculoskeletal painful conditions, but apparently not specific for chronic pain conditions.

It is possible to perform an additional meta-analysis on three articles to calculate the effects of exercise on T cell inflammatory markers IL-2 and IL-4 from three of the included articles [39,41,45]. However, since these studies were published by the same research team and examined similar population groups, concerns regarding publication bias and generalizability arise. Another three included articles shared similar characteristics of studying the effect of core exercise compared with general exercise using IL-6 as an outcome measure [40,49,50]. Unfortunately, two of them did not provide sufficient statistical data (i.e., mean ± SD) of their results. In Minobes-Molina et al. [50] both study and control groups followed similar indistinguishable back strengthening exercise programs. Therefore, we did not further analyze their results.

Regarding the risk of bias in the included studies, the majority presented some concerns, primarily due to insufficient reporting on allocation concealment during the randomization process. However, because this review focused on objective inflammatory biomarker outcomes, the risk of bias related to deviations from intended interventions and outcome measurement was minimized. The use of validated laboratory methods for biomarker assessment further reduced the potential influence of participant or personnel blinding on the results.

A minority of studies exhibited a high risk of bias which could compromise the reliability of findings in those cases. The study conducted by Ernberg et al. (2018) reported significant missing data for cytokine analyses, with 15 participants excluded from the exercise group and 18 from the relaxation group due to incomplete data [36]. In the study conducted by Uzunel et al. (2023), a substantial proportion of pain-associated biomarker data (e.g., IL-6, calcitonin gene-related peptide (CGRP), substance P) were missing, with 25–30% of samples either not analyzed or excluded due to technical issues. Additionally, dropout rates were 15% in the exercise group and 20% in the spinal orthosis group [51]. In Zhang et al.’s (2013) study, the study’s allocation process (based on admission order) also complicates the reliability of its findings in the presence of any unreported missing data [32].

The included studies utilized a range of biomarkers beyond inflammatory markers, including metabolic and growth factors (IGF-1, glucose, insulin, HOMA-IR) to assess metabolic changes, matrix and cartilage proteins (COMP, proteoglycan) to evaluate cartilage health, and pain and stress modulation markers (β-endorphin, cortisol) to assess pain relief and stress response. In addition, clinical outcomes were measured to capture broader effects of exercise interventions on pain and function. These included pain and functional scores (VAS, NRS, WOMAC, Roland–Morris Disability Questionnaire), physical function metrics (pain-free walking distance, step count, isokinetic torque), quality of life assessments (DAS28, RAID), and muscle and joint measures (multifidus muscle thickness, joint range of motion, morning stiffness duration). As these non-inflammatory markers and clinical outcomes were outside the scope of this meta-analysis, they were not included.

### Limitations

As this review specifically focused on biomarkers as outcome measures, the risk of bias assessment was tailored accordingly. It is important to note that the quality and risk of bias ratings of the included articles may differ if other clinical outcomes were considered. Notably, a significant number of included studies were conducted by Nambi’s research team [39,40,41,45,52]. While these studies were assessed as having a low risk of bias according to ROB 2, together they may introduce publication bias. Furthermore, the potential use of similar population subjects across these studies could limit the generalizability of the findings, as previously discussed. In addition, the majority of eligible studies, including those included in the meta-analysis, had small sample sizes. This may be attributed to the high cost of laboratory procedures required for biomarker measurements.

Another limitation of this review is that the meta-analyses were based primarily on short-term follow-up data (4–6 weeks), which may reflect early physiological responses to exercise rather than long-term adaptation effects. As such, the findings should be interpreted in the context of short-duration interventions, and future studies are needed to evaluate the sustained impact of exercise over longer periods. There was also variability in how included studies reported or controlled for the use of pain and anti-inflammatory medications. While many studies implemented exclusion criteria or required stable medication regimens, others lacked detailed reporting. As such medications may influence inflammatory biomarkers, this inconsistency could introduce potential confounding effects that were considered during the risk of bias assessment. The variability in biomarker measurements across different disease stages and due to varying laboratory techniques could introduce significant heterogeneity.

## 5. Conclusions

Our systematic review suggests that exercise training is effective in regulating the levels of inflammatory markers in patients with musculoskeletal pain. Specifically, isokinetic exercise appears to be particularly effective in reducing the acute-phase protein CRP. Exercise may also reduce the levels of TNF-α and IL-6, although the certainty of evidence for these outcomes is low due to inconsistency and imprecision. These findings suggest that future research on exercise interventions for musculoskeletal pain should consider systemic anti-inflammatory effects in addition to mechanical effects.

## Figures and Tables

**Table 1:** Characteristics of included studies.

Authors	Study Design	Country of Study	Main Diagnosis	Sample Size	Age and Gender Ratio	Inclusion Criteria	Exclusion Criteria
Melikoglu et al., 2006	RCT	Turkey	RA	40	Dynamic exercise group: 46.4 ± 8.3 years old ROM exercise group: 50.3 ± 9.7 years old Control group: 43.2 ± 6.9 years old All females	Diagnosed with RA according to the 1987 revised ACR criteria. Female participants only. Not clinically active disease, defined by morning stiffness duration > 30 min, six or more tender joints, three or more swollen joints, or ESR > 28 mm/h. Stable dosage of disease-modifying drugs in the last 3 months. Functional status class I–II as defined by the ACR.	Use of oral contraceptive drugs. Comorbid medical conditions known to affect IGF status. Inability to tolerate walking on treadmill. Sedentary healthy controls were selected according to a questionnaire quantifying physical activity undertaken during a normal week.
Bjersing et al., 2012	RCT	Sweden	FM	49	20 to 60 years old All females	Interested in exercising outdoors twice a week for 15 weeks. Able to perform a bicycle test at 50 watts.	Non-Swedish speakers/readers. Severe somatic or psychiatric diseases. Ongoing or planned physical therapy, including exercise. Inability to adhere to scheduled exercise sessions.
Simão et al., 2012	RCT	Brazil	KOA	32	Whole-body vibration group: 72.0 ± 7.4 years old; 2 males and 10 females Squat exercise group: 69.0 ± 3.7 years old; 1 male and 9 females Control group: 71.0 ± 5.3 years old; 1 male and 10 females	Patients aged 60 years or older with a diagnosis of KOA based on the ACR clinical and radiographic criteria.	Recent knee trauma, use of any locomotion device, physiotherapy or other rehabilitation in the last 3 months, absence of clinical and cognitive conditions for physical activities, glucocorticoid use for at least 2 months, orthopedic disease, neurological, respiratory, or acute cardiac issues, vestibular disorders, immunosuppression or immunodeficiency, lack of sphincter control, or cognitive deficits.
Sjøgaard et al., 2013	RCT	Denmark	Trapezius myalgia	28	Specific strength training group: 44.0 ± 9.8 years old General fitness training group: 44.0 ± 9.8 years old Healthy control group: 44.0 ± 9.1 years old All females	Females with trapezius myalgia employed in jobs with repetitive and monotonous work tasks, diagnosed based on pain or discomfort in the neck/shoulder region for more than 30 days in the last year, and clinical signs of palpable tenderness and tightness in the trapezius muscle.	Serious conditions such as previous trauma or injuries, life-threatening diseases, cardiovascular diseases, or arthritis in the neck and shoulder.
Zhang et al., 2013	RCT	China	KOA	100	Treatment group: 53.0 ± 5.6 years old; 20 males and 30 females Control group: 52.3 ± 7.0 years old; 18 males and 32 females	Diagnosed with KOA based on American Rheumatism Association criteria. Age 40–70 years. Disease classification of I-III by X-ray. Not receiving other treatment methods or medication.	Significant stenosis of the joint space or bone bridge connection between the joints (bony ankylosis). Disease classification IV by X-ray. Primary disease of the knee affecting joint structures. Active gastrointestinal diseases, cardiovascular, cerebrovascular, liver, kidney, or hematopoietic system diseases. Mental illness, pregnancy, lactation, and known allergy to diclofenac.
Delaney et al., 2015	RCT	Australia	Intermittent claudication	35	Experimental group: 73.4 ± 9.1 years old; 12 males and 5 females Control group: 69.4 ± 9.6 years old; 13 males and 4 females.	Clinical history consistent with intermittent claudication. ABPI < 0.9. Radiographic evidence of infra-inguinal disease without aorto-iliac disease	Diagnosis of critical limb ischemia. Recent (<12 months) peripheral vascular intervention (surgery or endovascular). Pre-existing cardio-respiratory conditions limiting exercise capacity.
Samut et al., 2015	RCT	Turkey	KOA	42	Isokinetic exercise: 62.5 ± 7.7 years old Aerobic exercise: 57.6 ± 5.8 years old Control group: 60.9 ± 8.9 years old All females	Postmenopausal women and men aged over 50 years. Diagnosed with KOA according to the ACR criteria. Kellgren–Lawrence grade 2–3 knee OA. Sedentary lifestyle (less than 60 min of moderate- to high-intensity activity per week).	Cooperation problems, depression, cognitive impairment, neurologic impairment/disease, orthopedic problems, or inflammatory arthritis. Regular exercise habits, physical therapy or intra-articular injection in the last three months, or cardiovascular problems. End-stage disease, immune-suppressive drug usage, infection or inflammatory condition, pregnancy, or malignant disease.
Sandstad et al., 2015	Randomized crossover design study	Norway	RA	18	High-intensity interval training group: 32.4 ± 8.3 years old Control group: 33.4 ± 8.5 years old All females	Women aged 20–50 years with RA or adult-onset JIA, stable disease with no changes in medication for the last 2 months.	Regular exercise training > 2 times a week before the study, participation in <80% of exercise sessions, heart disease, lung disease, pregnancy, lactation, active arthritis despite medication, or new synthetic or biologic DMARDs.
Ernberg et al., 2018	Randomized controlled multicenter trial	Sweden	FM	125	Progressive resistance exercise group: 51.2 ± 9.4 years old Relaxation therapy group: 51.2 ± 9.4 years old Healthy controls: 48.2 ± 11.4 years old All females	Women with FM diagnosed according to the ACR-1990 classification criteria. Working age (20–65 years).	High BP (>160/90 mmHg), osteoarthritis in the hip or knee, other severe somatic or psychiatric disorders, primary causes of pain other than FM, high alcohol consumption (audit > 6), participation in a rehabilitation program within the past year, regular resistance exercise or relaxation therapy inability to understand or speak Swedish, and inability to refrain from analgesics, NSAID, or hypnotics for 48 h prior to examinations.
Lau et al., 2019	RCT	Hong Kong	RA	21	Neural mobilization group: 57.5 ± 7.1 years old Control group: 57.5 ± 7.1 years old All females	Patients fulfilling the ACR 1987 revised criteria for rheumatoid arthritis. Non-smokers.	Not explicitly listed in the visible text but generally includes those who cannot follow the exercise protocol or have other contraindications to exercise.
Nadi et al., 2019	Quasi-experimental study	Iran	Diabetic neuropathy	45	Exercise for peripheral neuropathy group: 55.46 ± 2.47 years old; Resistance group: 56.13 ± 3.39 years old Control group: 54.80 ± 3.29 years old All females	Female sex, age range 50–60 years, minimum diabetes history of 7 years, HbA1c ≥ 6.5%, mild-to-moderate diabetic neuropathy, and fasting blood glucose ≥ 156 mg/dL.	Serious diseases (autoimmune, liver, renal, thyroid, cerebrovascular injury, fractures, tumor), voluntary withdrawal, irregular meeting attendance, failure to complete tests, weight change > 3 kg during intervention, other diabetes complications, medication change, anti-inflammatory drug use, and blood glucose imbalance before training.
Minobes-Molina et al., 2020	RCT	Spain	Non-specific LBP	39	Traditional trunk exercise group: 50.9 ± 11.0 years old Specific stabilization exercise group: 50.1 ± 9.8 years old All females	Females aged between 18 and 70 years. Diagnosed with non-specific LBP (fewer than 6 weeks of pain duration) by a specialist doctor. Imaging used to rule out other spinal disorders. Not under pharmacological treatment for pain.	Diagnosed with other spinal disorders. Any serious co-morbidities (e.g., cancer, severe lung pathology). Inability to perform exercises. Presence of cognitive impairment. Having followed a specific training program with a physiotherapist in the previous three months. Having been treated with analgesic infiltration in the previous 6 weeks. Failure to follow their 20-treatment schedule exactly.
Nambi, Abdelbasset, Elsayed, et al., 2020	RCT	Saudi Arabia	PTOA following ACL injury	60	Virtual reality training group: 22.8 ± 1.3 years old Sensory motor training group: 22.6 ± 1.4 years old Control group: 21.9 ± 1.3 years old All males	Male football players, age 18–25 years. Chronic (≥3 months) PTOA following ACL injury. Pain rating 4–8 on VAS.	Other orthopedic, neural, systemic, or psychological conditions, awaiting surgery, previous treatment or physical training, and unwillingness to participate.
Nambi et al., 2020	RCT	Saudi Arabia	Chronic (≥3 months) OA after ACL injury	60	Isokinetic training group: 22.3 ± 1.2 years old Sensory motor training group: 22.4 ± 1.5 years old Control group: 22.9 ± 1.7 years old All males	University football players. Age between 18 and 25 years. Male players. Chronic (≥3 months) OA after ACL injury. Pain intensity between 4 and 8 on VAS	Severe musculoskeletal, neural, somatic, or psychiatric conditions. Awaiting surgery. Alcohol or drug abuse. Involvement in other weight and balance training programs. Other soft-tissue injuries, fracture of the lower limbs and pelvic bone, or deformities.
Nambi, Abdelbasset, Alsubaie, et al., 2020	RCT	Saudi Arabia	Chronic LBP	60	Isokinetic training group: 22.1 ± 1.8 years old Core stabilization training group: 22.3 ± 1.7 years old Control group: 21.9 ± 1.8 years old All males	University male football players aged 18–25 years. Chronic (≥3 months) LBP. Pain intensity between 4 and 8 on VAS. Diagnosed with LBP by an orthopedic surgeon and referred for physical therapy.	Severe musculoskeletal, neural, somatic, and psychiatric conditions. Other soft tissue injuries, fractures of the lower limbs and pelvic bone, or deformities. Awaiting spine surgery. Alcohol or drug abuse. Involvement in other weight and balance training programs.
Oğuz et al., 2021	RCT	Turkey	KOA	22	Exercise training group: 51.0 ± 3.7 years old Exercise training + kinesio taping group: 48.2 ± 7.6 years old All females	Female patients, aged 38–60 years, diagnosed with KOA according to the ACR criteria, and classified as Kellgren–Lawrence index II and III.	RA, severe organ failure, previous joint replacement, osteoporosis, and diagnosis of any disease that could limit performance.
Park et al., 2021	RCT	South Korea	KOA	75	Isometric exercise + electromyostimulation group: 65.7 ± 3.2 years old Isometric exercise group: 66.9 ± 4.6 years old Control group: 68.0 ± 4.2 years old All females	Females aged over 60 years. Diagnosed with early KOA (Grade I or II) based on bilateral radiographic examination. Irregular exercise habits for the previous six months. No medications, including steroids or intra-articular injections within the last three months.	Deformities of the knee, hip, or back. Central or peripheral nervous system involvement. Use of pacemakers or internal metallic materials. History of impairment of a major organ system or psychological disorders.
Du et al., 2022	RCT	China	RA	20	Experimental group: 43.8 ± 3.57 years old; 8 females and 2 males Control group: 52.4 ± 3.35 years old; 8 females and 2 males	Age between 30 and 65 years. Diagnosed with RA using the ACR/EULAR 2010 classification criteria. Low-to-moderate Disease Activity Score in 28 joints (2.6 < DAS28 < 5.1). Stable medical treatment (no alternative medical treatments during study period). No participation in structured exercise for the preceding 3 months.	Heart or kidney disease and other pre-existing medical conditions that might prevent participation in the exercise program.
Jayabalan et al., 2022	Two-phase crossover sequential design	US	KOA	10	KOA participants: 67.5 ± 5.3 years old; 7 males and 3 females Control participants: 62.2 ± 3.0 years old; 3 males and 2 females	Participants with symptomatic unilateral medial KOA meeting ACR clinical criteria. Regular golfers (playing at least 1–2 times per month).	Previous total knee arthroplasty or other lower limb joint pain (hips or ankles) or LBP. Severe cardiovascular disease, uncontrolled hypertension, or stroke. Participants who typically require pain medications during a round of golf.
Nambi et al., 2023	RCT	Saudi Arabia	CNLBP	60	Virtual reality exercise group: 23.2 ± 1.6 years old Isokinetic exercise group: 22.9 ± 1.7 years old Conventional exercise group: 22.8 ± 1.8 years old All males	Male soccer players aged 18–25 years. CNLBP for three or more months. Pain score ranging from 4 to 8 on 10 cm VAS.	Lumbar stenosis, lumbar radiculopathy, lumbar spondylolisthesis, or spinal injuries. Serious pathologies of the thoracolumbar spine. Associated low-back muscle and tendon injuries or fracture of the pelvic bone and lower extremity bones. Spine dysfunctions, awaiting spine surgery. Participants taking steroids, medications, or analgesics. Serious pathologies of the thoracolumbar spine. Participating in other resistance training and physical training programs.
Oghumu et al., 2023	RCT	Nigeria	CNLBP	54	Lumbar stabilization exercise group: 49.5 ± 7.8 years old; 11 males and 15 females Graded activity exercise group: 49.5 ± 7.8 years old; 11 males and 15 females Control group: 46.5 ± 6.1 years old; 15 males and 12 females	Patients with CNLBP referred for physiotherapy, aged 18–60 years, with or without radiating symptoms for at least three months.	Spinal inflammatory disease, history of spinal fracture/dislocation, motor/sensory deficit, pregnancy, systemic or medical conditions like diabetes, hematological disorders, acute/chronic liver diseases, autoimmune diseases, use of pain medications within the last two days, unwillingness to stop analgesics, history of psychotropic medications, open wounds, and history of smoking/drinking in the last six months.
Uzunel et al., 2023	RCT	Sweden	Back pain and self-reported osteoporosis	113	Exercise group: 77.7 ± 8.6 years old Spinal orthosis group: 78.0 ± 8.7 years old Control group: 72.9 ± 7.9 years old All females	Women aged 60–93 years. Self-reported osteoporosis. Experiencing back pain with or without vertebral fractures.	Inability to follow the research protocol. Insufficient Swedish language skills. Diagnosed with spinal stenosis.
Roberts et al., 2024	RCT	UK	axSpA	20	Exercise group: 47.1 ± 11.4 years old; 5 males and 5 females Control group: 43.6 ± 10.1 years old; 7 males and 3 females	Diagnosed with axSpA, on stable NSAID dose, no heart disease or anemia, not pregnant/planning pregnancy, able to meet study demands, and low habitual physical activity.	History of heart disease, high physical activity level (≥1 h moderate activity per day), or inability to walk.

Abbreviation Legend: RCT—randomized controlled trial; JIA—Juvenile Idiopathic Arthritis; RA—rheumatoid arthritis; KOA—knee osteoarthritis; axSpA—axial spondyloarthritis; CNLBP—chronic non-specific low-back pain; FM—fibromyalgia; OA—osteoarthritis; LBP—low-back pain; PTOA—Post-Traumatic Osteoarthritis; ACL—Anterior Cruciate Ligament; ACR—American College of Rheumatology; ACR/EULAR—American College of Rheumatology/European League Against Rheumatism; ABPI—Ankle–Brachial Pressure Index; DAS28—Disease Activity Score in 28 Joints; BP—blood pressure; ESR—erythrocyte sedimentation rate; HbA1c—hemoglobin A1c; NSAID—non-steroidal anti-inflammatory drug; DMARDs—disease-modifying anti-rheumatic drugs; bDMARDs—biologic disease-modifying anti-rheumatic drugs; KL grade—Kellgren–Lawrence Grade; VAS—Visual Analog Scale; WOMAC—Western Ontario and McMaster Universities Osteoarthritis Index; MHAQ—Modified Health Assessment Questionnaire; BMI—body mass index; QUALEFFO-41—Quality of Life Questionnaire for Osteoporosis; SF-36—Short Form (36) Health Survey; MCS/PCS—Mental/Physical Component Summary of SF-36.

**Table 2:** Outcome measures and follow-up periods of included studies.

	Intervention Group	Comparison 1	Comparison 2	Outcome Measures			Follow-Up Period
Authors				Inflammatory Biomarkers	Non-Inflammatory Biomarkers	Other Outcomes	
Melikoglu et al., 2006	Dynamic exercise program on a treadmill, maintaining heart rate at 60% of age-predicted maximum heart rate. Five sessions per week for two weeks. Duration of each session: 20 min.	ROM exercises for all joint movements of the upper and lower extremities, performed actively at a low pace. Exercise frequency: Five sessions per week for two weeks. Duration of each session: 20 min.	Nil	CRP, ESR	IGF-1, IGFBP-3	VAS, morning stiffness duration, HAQ, RAI	15 days
Bjersing et al., 2012	Engaged in a moderate- to high-intensity Nordic walking program, conducted twice a week for 40 to 45 min over 15 weeks.	Engaged in supervised low-intensity walking, conducted twice a week for 40 to 45 min over 15 weeks.	Nil	IL-6, IL-8, SP, NPY, MMP-3	IGF-1	Pain score, 6MWT	15 weeks, 30 weeks
Simão et al., 2012	Squat Exercises + Whole-Body Vibration: 12 weeks, 3 times per week. Squats were performed on a vibratory platform with a 10–60° knee flexion range, maintaining 3 s of isometric contraction at 60° flexion.	Squat Group: Participants performed the same squat exercises as the platform group but without vibration, for the same duration and frequency.	Control Group: Participants did not receive any training and were instructed not to change their lifestyle during the study.	sTNFR1, sTNFR2	NA	WOMAC, Berg Balance Scale, gait speed test, 6MWT	12 weeks
Sjøgaard et al., 2013	Specific Strength Training: 10 weeks, 3 times per week, 20 min per session. Performed 5 different dumbbell exercises targeting the neck and shoulder muscles (1-arm row, shoulder abduction, shoulder elevation, reverse flies, upright row). Progression from 12 repetitions maximum (RM) (~70% of max) to 8 RM (~80% of max).	General Fitness Training: 10 weeks, 3 times per week, 20 min per session of cycling on a Monark ergometer at 50–70% of VO2max, with progressive intensity increase.	Reference Group: Participants attended weekly health counseling sessions (~1 hr per week) on workplace ergonomics, diet, general health, relaxation, and stress management, but did not perform any structured physical exercise.	IL-6, TNF-α	IGF-1, HSP72, HSc70	Pain intensity, muscle metabolic adaptations (Glut4, GS, PDH-E1α, CytC, HKII), functional capacity	10 weeks
Zhang et al., 2013	Exercise Therapy: 4 weeks, 4 days per week. Sessions included knee joint flexion and extension, followed by isometric quadriceps contractions at 0° and 90° knee flexion. Each contraction lasted 10 s, followed by 10 s relaxation, repeated 10 times per session, with 5 sets per angle and 1 min rest between sets. Performed in a sitting position to avoid weight-bearing stress. Participants also received oral diclofenac sodium (75 mg, twice daily).	Control Group: Participants received only oral diclofenac sodium (75 mg, twice daily) without exercise intervention.	Nil	TNF-α, hs-CRP, MMP-3	NA	Knee function index score, pain intensity, synovial fluid cytokine levels	4 weeks
Delaney et al., 2015	Treadmill-based SET, participants walked until claudication pain became unbearable, rested until pain resolved, repeated cycle for the session duration	Combination-based SET, three sets of 8–12 repetitions of hamstring curls, seated calf press, leg press, knee extension, and hip abduction/adduction, followed by walking on the treadmill until onset of claudication pain, rest until pain resolved, cycle repeated for session duration.	Nil	NO, ADMA	NA	PFWD, FMD, RHI, QoL	12 weeks
Samut et al., 2015	Isokinetic Exercise: 6 weeks, 3 days per week. Training included concentric flexion and extension exercises at angular velocities of 60°/s, 90°/s, 120°/s, and 180°/s using a Biodex Isokinetic System. One set of contraction in the first session was increased by 1 set per session until reaching 6 sets. Rest periods: 20 s between sets, 2 min between legs.	Aerobic Exercise: 6 weeks, 3 days per week on a treadmill. Training intensity was set at 65–70% of age-related heart rate for the first 4 weeks, then increased to 70–75% for the last 2 weeks. Sessions included a 5 min warm-up and 5 min cool-down.	No Intervention: Participants were informed about knee osteoarthritis and given recommendations, but no exercise intervention was provided.	IL-6, TNF-α, CRP	NA	VAS, WOMAC, functional capacity (6MWT), muscle strength (30 s sit-to-stand test)	6 weeks
Sandstad et al., 2015	High-Intensity Interval Training: 10 weeks, 2 times per week, performed on spinning bikes. Each session consisted of 4 × 4 min high-intensity intervals at 85–95% of HRmax, interspersed with 3 min recovery periods at ~70% of HRmax. Each session started with a 10 min warm-up and lasted a total of 35 min.	Participants continued their usual lifestyle without structured exercise.	Nil	CRP, PTX3	IGF-1, COMP	VAS, DAS28, body composition (BMI, body fat percentage, muscle mass), cardiovascular fitness (VO2max, 1 min heart rate recovery), functional disability (MHAQ)	10 weeks
Ernberg et al., 2018	15 weeks, twice per week, supervised by trained physical therapists, 10 min of bicycling warm-up, 50 min strength training focusing on lower extremities, initial loads at 40% MVC progressing to 70–80% MVC.	15 weeks, twice per week, supervised by experienced physiotherapists, autogenic training including relaxation and autosuggestion, followed by stretching exercises, approximately 25 min per session.	Nil	IL-2, IL-6, IL-8, IL-17A, TNF-α, IP-10, Eotaxin	NA	Pain, 6MWT, PPT, FIQ, PDI, SF-36	15 weeks
Lau et al., 2019	Participants performed neural mobilization exercises targeting the median nerve, musculocutaneous nerve, femoral nerve, saphenous nerve, and the entire nervous system. The exercises were performed 10 times per set, 2 sets per day for 4–8 weeks.	Participants performed gentle joint mobilization exercises for the fingers, wrist, elbow, shoulder, spine, and lower limbs without touching the end range of joints and stressing the nervous system. Exercises were performed 10 times per set, 2 sets per day for 4–8 weeks.	Nil	ESR	NA	RAID score, pain, coping (self-efficacy)	4–8 weeks
Nadi et al., 2019	Resistance Group: 12 weeks, three sessions per week, exercises with 30% repetition maximum including leg press, arm curls, military press, push-ups, squats, knee extensions, heel raises, back extensions, knee sit-ups, and upright rowing.	EPN Group: 12 weeks, three sessions per week, 12 lower extremity movements including hamstring stretching, knee swirling, gradual stretching of the sciatic nerve, stretching of the leg muscles, and ankle range movements exercises.	Control Group: Continued with their daily activities without any specific intervention.	TNF-α, IL-10, CRP	FBG, HbA1c	Pain, tingling, static and dynamic balance (DEMMI), MNSI	12 weeks
Minobes-Molina et al., 2020	Traditional Trunk Exercise Program (TTEP): 20 sessions, 3–5 times per week, first 5 sessions with infrared lamp and TENS, followed by 15 sessions with 10 exercises focusing on global abdominal and back muscles.	Specific Stabilization Exercise Program (SSEP): 20 sessions, 3–5 times per week, first 5 sessions with infrared lamp and TENS, followed by 15 sessions with 10 exercises focusing on transversus abdominis, multifidus, and internal oblique muscles.	Nil	IL-6, TNF-α	NA	VAS, disability (RMDQ)	1 month
Nambi, Abdelbasset, Elsayed, et al., 2020	Virtual Reality Training: 4 weeks, 20 min per session, two sessions per day, 5 days per week. Training involved the use of the Pro-Kin system PK 252 N focusing on knee muscle improvement. Exercises included virtual tasks such as shooting balls by moving the knee joint in different directions.	Sensory Motor Training: 4 weeks, 5 repetitions per set for 3 sets, 3 min rest between sets, 5 days per week. Exercises included static standing on hard and foam plates, single-leg standing with closed eyes, semi knee bending, dynamic forward kicking, T-band kicking, toe jumping, heel jumping, and bilateral and unilateral squatting.	Control Group: Supervised conventional exercise program for knee muscles, including quadriceps, hamstrings, glutei, and calf muscles. Exercises performed 10–15 repetitions per set for 3 sets, 1 min rest between sets, 5 days per week, with stretching exercises for each muscle group.	CRP, TNF-α, IL-2, IL-4, IL-6	NA	VAS, WOMAC score	4 weeks, 8 weeks, 3 months
Nambi et al., 2020	Isokinetic Training Group: 4 weeks, exercises performed at angular speeds of 60, 90, and 120 degrees/s, with 15 repetitions per set for 3 sets, 5 days per week. Training involved using an isokinetic dynamometer to perform knee flexion and extension exercises.	Sensory Motor Training Group: 4 weeks, exercises performed in three stages (static, dynamic, and functional), with 5 repetitions per set for 3 sets, 5 days per week. Exercises included static standing on firm and soft surfaces, single-leg standing with eyes closed, knee bends, forward stepping thrusts, T-band kicks, toe skipping, heel skipping, and squats.	Control Group: Home-based exercises including stretching and strengthening exercises for quadriceps, hamstrings, glutei, and calf muscles. Exercises performed 10–15 repetitions/day, 5 days a week, for 4 weeks.	CRP, TNF-α, IL-2, IL-4, IL-6	NA	VAS, WOMAC score	4 weeks, 8 weeks, 6 months
Nambi, Abdelbasset, Alsubaie, et al., 2020	Isokinetic Training: 4 weeks, 5 days/week. Training on an isokinetic dynamometer (Biodex, New York, NY, USA) in a standing position with trunk flexion/extension at 60°, 90°, and 120°/s, 15 reps per set, 3 sets per session. Participants were stabilized using Velcro straps to prevent compensatory movements.	Core Stabilization Training: 4 weeks, 5 days/week. Exercises performed on Swiss ball (supine bridge, sit-up, arms-legs cross lifting, side bridge). Participants performed 3 sets of 10 reps, holding positions for 10 s with 3 s rest between.	Conventional Exercise: 4 weeks, 5 days/week. Focused on isometric and isotonic core exercises for abdominal and back muscles, performed at 10–15 reps/set for 3 sets. Stretching (3 reps, 10 sec) for hamstrings, hip flexors, and lumbar extensors.	CRP, TNF-α, IL-2, IL-4, IL-6, IL-8	NA	VAS, CSA of paraspinal muscles (Psoas major, Quadratus lumborum, Multifidus, Erector spinae)	4 weeks
Oğuz et al., 2021	Isokinetic Training Group: 4 weeks, performed at angular speeds of 60, 90, and 120 degrees/s, with 15 repetitions per set for 3 sets, 5 days per week. Training involved using an isokinetic dynamometer to perform trunk flexion and extension exercises.	Graded Activity Exercise Group: Focused on increasing activity tolerance through individualized sub-maximal exercises with cognitive behavioral principles. The intervention was also conducted over 10 weeks, with sessions twice a week. Exercises included isometric strengthening, bicycle ergometry, and cognitive behavioral therapy.	The control group received no intervention and served as a baseline for comparisons.	MMP-1, MMP-3	COMP	VAS, WOMAC, functional status	6 weeks
Park et al., 2021	Isometric Exercise + Whole-Body Electromyostimulation (WB-EMS): 8 weeks, 3 days per week, 30 min per session. Exercises included abdomen crunches, bridge, leg raises, side planks, back extension, front planks, front lunges, and squats. Performed in an alternation of a 6 s contraction with a 4 s break. The WB-EMS suit was used with stimulation settings (85 Hz frequency, 350 µs impulse width).	Isometric exercise: performed the same isometric exercises while wearing a WB-EMS suit without electrical stimulation provided.	Control group did not perform exercises but participated in meditation and light stretching for 30 min while wearing the WB-EMS suit without stimulation.	IL-6, TNF-α, CRP, Resistin	NA	VAS, KOOS, muscle strength, body composition (fat mass, fat percentage, skeletal muscle mass)	8 weeks
Du et al., 2022	A 12-week training program with 50 min tai chi movements and 20 min hand exercises. One session per week with a professional instructor and daily practice at home.	Participants maintained their usual lifestyle without any special exercise intervention.	Nil	CRP, ESR, RF	NA	DAS28, TJC, SJC, morning stiffness, HAQ, SDS, SAS	12 weeks
Jayabalan et al., 2022	A 12-week exercise program consisting of two sessions per week. Each session included 40 min of progressive resistance training to improve muscle strength, joint mobility, and overall physical function.	The control group did not participate in the exercise program and continued their usual activities without any additional intervention.	Nil	TNF-α, IL-1β, IL-6, MMP-3, MMP-13	COMP	Pain, step count, RPE, heart rate, MVPA duration	Same day post intervention measurement
Nambi et al., 2023	Virtual Reality Exercise: 4 weeks, 30 min/session, 5 days/week. Training with the Pro-Kin Techno Body system using a car race game, where participants moved their trunk in different directions to control the game. Resistance increased by modifying road steepness, terrain, and number of opposing cars.	Isokinetic Exercise: 4 weeks, 30 min/session, 5 days/week. Training with Humac Norm (Stoughton, MA, USA) isokinetic dynamometer. Participants performed trunk flexion and extension at 45°, 60°, and 90°/s while standing upright, with Velcro stabilization to isolate trunk movements.	Conventional Exercise: 4 weeks, 30 min/session, 5 days/week. Performed routine core and balance exercises targeting abdominal, and back muscles. Included 10–15 repetitions per set with 3 sets per session and stretching exercises for the lower limb muscles.	CRP, TNF-α, IL-2, IL-4, IL-6	NA	VAS, CSA of paraspinal muscles (Psoas major, Quadratus lumborum, Multifidus, Erector spinae)	4 weeks
Oghumu et al., 2023	Lumbar Stabilization Exercise: 10 weeks, 2 sessions per week. Core-focused exercises targeting transversus abdominis, multifidus, pelvic floor, and diaphragm.	Graded Activity Exercise: 10 weeks, 2 sessions per week. Based on time-contingency and cognitive–behavioral principles. Activities included isometric strength training (quadriceps, hamstrings, glutes, back extensors), cycling ergometry, and proprioceptive training. Pain tolerance was reinforced with behavioral modification strategies.	Health control group with no intervention	IL-1A, IL-6, IL-18R1, IL-18RAP, COX-2	NA	VAS, disability (RMDQ), catastrophizing, diverting attention, cognitive coping, pain reinterpretation	10 weeks
Uzunel et al., 2023	Exercise Group: 6 months, 1 h supervised group exercise per week, led by a physiotherapist. The program included gym machines, resistance bands, balance plate, and Bobath ball exercises focusing on back extensor and shoulder muscle strength, leg muscle strength, balance, and posture. Participants also performed a home exercise program at least 4 times per week.	Spinal Orthosis Group: Wore the activating Spinomed spinal orthosis for 10 min per day initially, increasing gradually to at least 2 h per day. The orthosis provided feedback to activate back extensor muscles.	Control Group: Participants were instructed to continue with their usual lifestyle without structured exercise or orthosis use.	IL-6	NA	VAS, QUALEFFO-41, SF-36, spinal orthosis effects, functional mobility	6 months
Roberts et al., 2024	Platform Group: Participants performed squat exercises on a vibratory platform 3 times per week, on alternate days, for 12 weeks. The exercise involved squatting from approximately 10° to 60° of knee flexion, with standardized time and verbal encouragement for consistency. The vibration parameters were 35–40 Hz frequency, 4 mm amplitude, and 2.78–3.26 g acceleration.	Control Group: Daily oral diclofenac sodium (75 mg twice daily) without kinesitherapy.		IL-6, CRP, TNF-α, IL-17	NA	Spinal pain, body fat percentage, waist circumference, blood pressure (systolic and diastolic), physical function (sit-to-stand tests), BASDAI, BASFI, WPAI, ASAS-HI	12 weeks

Abbreviation Legend: Inflammatory Biomarkers: ADMA—Asymmetric Dimethylarginine; CRP—C-reactive protein; ESR—erythrocyte sedimentation rate; hs-CRP—high-sensitivity C-reactive protein; IL—interleukin; IP-10—Interferon Gamma-Induced Protein 10; MMP—matrix metalloproteinase; NPY—Neuropeptide Y; PTX3—Pentraxin 3; RF—Rheumatoid Factor; SP—Substance P; sTNFR1/2—Soluble Tumor Necrosis Factor Receptor 1/2;TNF-α—Tumor Necrosis Factor-alpha. Non-inflammatory Biomarkers: COMP—Cartilage Oligomeric Matrix Protein; CytC—Cytochrome C; FBG—Fast Blood Glucose; GH—Growth Hormone; Glut4—Glucose Transporter Type 4; GS—Glycogen Synthase; HbA1c—Glycated Hemoglobin; HKII—Hexokinase II; HOMA-IR—Homeostatic Model Assessment for Insulin Resistance; HSc70—Heat Shock Cognate Protein 70; HSP72—Heat Shock Protein 72; IGF-1—Insulin-like Growth Factor 1; IGFBP-3 –Insulin-like Growth Factor Binding Protein-3; KS—Keratan Sulfate; PEA—Palmitoylethanolamide; PG—Total Proteoglycan; PRL—Prolactin; SEA—Stearoylethanolamide. Other Outcomes: ASAS-HI—Assessment of SpondyloArthritis International Society Health Index; BASDAI—Bath Ankylosing Spondylitis Disease Activity Index; BASFI—Bath Ankylosing Spondylitis Functional Index; CSA—Cross-Sectional Area; DAS28—Disease Activity Score in 28 Joints; DEMMI—De Morton Mobility Index; FIQ—Fibromyalgia Impact Questionnaire; FMD—Flow-Mediated Dilation; KOOS—Knee Injury and Osteoarthritis Outcome Score; MHAQ—Modified Health Assessment Questionnaire; MNSI—Michigan Neuropathy Screening Instrument; MVPA—moderate/vigorous physical activity; NRS—Numeric Rating Scale; PDI—Pain Disability Index; PFWD—pain-free walking distance; PPT—Pressure Pain Threshold; QoL—quality of life; RAID—Rheumatoid Arthritis Impact of Disease; RHI—Reactive Hyperemia Index; RPE—Rating of Perceived Exertion; SAS—Self-Rating Anxiety Scale; SDS—Self-Rating Depression Scale; SF-36—Short Form (36) Health Survey; MCS/PCS—Mental/Physical Component Summary of SF-36; TJC—Tender Joint Count; TSK-17—Tampa Scale for Kinesiophobia; VAS—Visual Analogue Scale; VO2max—Maximal Oxygen Consumption; WOMAC—Western Ontario and McMaster Universities Osteoarthritis Index; WPAI—Work Productivity and Activity Impairment Questionnaire; 6MWT—6-Minute Walk Test; MVC—Maximum Voluntary Contraction; HRmax—maximum heart rate; RMDQ—Roland–Morris Disability Questionnaire.

**Table 3:** GRADE recommendations.

Certainty Assessment							№ of Patients (Musculoskeletal Pain)		Effect	Certainty
№ of Studies	Study Design	Risk of Bias	In-consistency	In-directness	Imprecision	Other Considerations	Isokinetic Exercises	General Exercises	Absolute (95% CI)	
CRP (follow-up: range 4 weeks to 6 weeks)										
3	randomized trials	not serious	not serious	not serious	not serious	none	52	53	MD **0.4 mg/L lower** (0.44 lower to 0.36 lower)	⨁⨁⨁⨁ High
TNF (follow-up: range 4 weeks to 6 weeks)										
3	randomized trials	not serious	serious ^a^	not serious	serious ^b^	none	52	53	MD **4.24 pg/mL lower** (5.13 lower to 3.36 lower)	⨁⨁ Low ^a,b^
IL-6 (follow-up: range 4 weeks to 6 weeks)										
4	randomized trials	not serious	serious ^a^	not serious	serious ^c^	none	73	73	MD **1.59 pg/mL lower** (2.61 lower to 0.56 lower)	⨁⨁ Low ^a,c^

CI: confidence interval; MD: mean difference. a. Considerable heterogeneity. b. The sample size is small, and some studies have CIs close to null, introducing uncertainty in effect size. c. Wide CIs, including both significant benefit and no effect, reduce confidence in the estimate. Considerable heterogeneity (I^2^ = 97%) and small sample size. ⨁, level of certainty.

## Data Availability

The data that support the findings of this study are available on reasonable request from the corresponding authors. It is a systematic review and does not involve primary patient data.

## Appendix A. Search Strings and Databases

Medline (via PubMed)

(((pain) AND ((Hip) OR (Knee) OR (Ankle) OR (Shoulder) OR (Wrist) OR (Hand) OR (Spine) OR (Back) OR (Neck) OR (Arthritis) OR (Musculoskeletal) OR (Limb) OR (Tendon)))

AND

((“Exercise”[MeSH]) OR (Aerobic) OR (“Resistance Training”[MeSH]) OR (Combined Training) OR (“Physical Activity”) OR (“Exercise Therapy”[MeSH]) OR (“Physical Exertion”[MeSH]) OR (Training) OR (Muscle Strengthening) OR (Weight Lifting) OR (Endurance Training) OR (Strengthening Programme)))

AND

((“C-reactive protein”[MeSH]) OR (Interleukin) OR (Tumor Necrosis Factor) OR (“Biomarkers”[MeSH]) OR (“Inflammatory marker”) OR (“Biological marker”) OR (“Inflammation”[MeSH]) OR (“Cytokines”[MeSH]) OR (Serum) OR (Plasma) OR (Saliva))

CINAHL (via EBSCOhost)

S1: pain

S2: hip OR knee OR ankle OR shoulder OR wrist OR hand OR spine OR back OR neck OR arthritis OR musculoskeletal OR limb

S3: tendon

S4: S2 OR S3

S5: exercise OR aerobic OR resistance training OR combined training OR physical activity OR exercise therapy OR physical exertion OR training OR muscle strengthening OR weight lifting OR endurance training OR strengthening programme

S6: c-reactive protein OR interleukin OR tumor necrosis factor OR biomarker OR inflammatory marker OR biological marker OR inflammation OR cytokine OR serum OR plasma OR saliva

S7: S1 AND S4 AND S5 AND S6

Web of Science (WOS)

#1: (“pain”)

#2: (“hip” OR “knee” OR “ankle” OR “shoulder” OR “wrist” OR “hand” OR “spine” OR “back” OR “neck” OR “arthritis” OR “musculoskeletal” OR “limb” OR “tendon”)

#3: (“exercise” OR “aerobic” OR “resistance training” OR “combined training” OR “physical activity” OR “exercise therapy” OR “physical exertion” OR “training” OR “muscle strengthening” OR “weight lifting” OR “endurance training” OR “strengthening programme”)

#4: (“C-reactive protein” OR “interleukin” OR “tumor necrosis factor” OR “biomarker” OR “inflammatory marker” OR “biological marker” OR “inflammation” OR “cytokine” OR “serum” OR “plasma” OR “saliva”)

#1 AND #2 AND #3 AND #4

Limited to articles only

Cochrane Library

pain AND Hip OR Knee OR Ankle OR Shoulder OR Wrist OR Hand OR Spine OR Back OR Neck OR Arthritis OR MSK OR Musculoskeletal OR Limb OR Tendon AND Exercise OR Aerobic OR Resistance Training OR Combined Training OR Physical Activity OR Exercise Therapy OR Physical Exertion OR Training OR Muscle Strengthening OR Weight Lifting OR Endurance Training OR Strengthening Programme AND C-Reactive Protein OR Interleukin OR Tumor Necrosis Factor OR Biomarker OR Inflammatory Marker OR Biological Marker OR Pro-inflammatory Marker OR Inflammation OR Cytokine OR Serum OR Plasma

limited to Trials
